# Relationship between the morphology of osteophytes and cartilage lesions in anterior ankle impingement in athletes: a cross-sectional study

**DOI:** 10.1186/s13047-023-00633-z

**Published:** 2023-05-31

**Authors:** Hiroki Yabiku, Tomohiro Matsui, Takeshi Sugimoto, Yasuyoshi Mase, Kotaro Higa, Fuminari Uehara, Takashi Toma, Chinatsu Azuma, Yasunori Tome, Kotaro Nishida, Tsukasa Kumai

**Affiliations:** 1 Department of Orthopedic Surgery, Graduate School of Medicine, University of the Ryukyus, 207 Uehara, Okinawa 903-0215 Nishihara, Japan; 2 Department of Orthopaedic Surgery, Saiseikai Nara Hospital, Nara, 4-643 Japan; 3 Department of Orthopedic Surgery, Osaka Global Orthopedic Hospital, 6-15-30 Sekime, Osaka Joto-ku, Osaka, Japan; 4 Department of Orthopedic Surgery and Rehabilitation, Hachioji Sports Orthopedic Clinic, 5-1 Nakacho, Tokyo Hachioji, Japan; 5 Faculty of Sports Sciences, Waseda University, 2-579-15 Mikajima, Saitama Tokorozawa, Japan

**Keywords:** Ankle impingement, Arthroscopy, Athlete, Cartilage, Sports, Tram-track lesion

## Abstract

**Background:**

The present study aimed to describe the frequency and severity of tram-track lesions in anterior ankle impingement in athletes and to evaluate the association between osteophyte morphology and severity of tram-track lesions, the distinctive cartilage lesions associated with tibial osteophytes in anterior ankle impingement syndrome.

**Methods:**

We evaluated 34 athletes who underwent arthroscopic osteophyte resection for anterior ankle impingement between January 2017 and March 2021.

**Results:**

We found tram-track lesions in 26 athletes (76.5%). Arthroscopic findings revealed the distribution of the International Cartilage Repair Society grades of tram-track lesions (grade 0, eight; grade 1, seven; grade 2, ten; grade 3, nine; grade 4, zero). These findings indicate that athletes with anterior ankle impingement syndrome may have more severe cartilage lesions than non-athletes. There was a positive correlation between the International Cartilage Repair Society grade and osteophyte size (r = 0.393, *p* = 0.021). We divided athletes into two groups according to the presence or absence of osteophyte protrusion into the joint space. Osteophyte protrusion was present in 14 athletes (41.2%). All athletes in the protrusion-type group had tram-track lesions; seven (50%) had International Cartilage Repair Society grade 3. The protrusion-type group’s International Cartilage Repair Society grade was significantly higher than that of the non-protrusion-type group (*p* = 0.008). The osteophyte sizes in the two groups were not significantly different (*p* = 0.341).

**Conclusions:**

Based on these findings, osteophyte protrusion should be assessed when an indication of arthroscopic treatment for anterior ankle impingement syndrome is considered, particularly in athletes.

## Background

Anterior ankle impingement syndrome is a clinical entity characterized by dorsiflexion restriction and chronic anterior ankle pain. These symptoms are caused by the pathological contact of bone and soft tissue structures in the anterior part of the ankle joint [1]. It is prevalent in athletes and decreases their athletic performance [2]. Anterior ankle impingement syndrome was first described by Morris in 1943 [3], followed by McMurray [4], and they termed anterior ankle impingement syndrome as athlete’s ankle and footballer’s ankle, respectively.

**Fig. 1:**
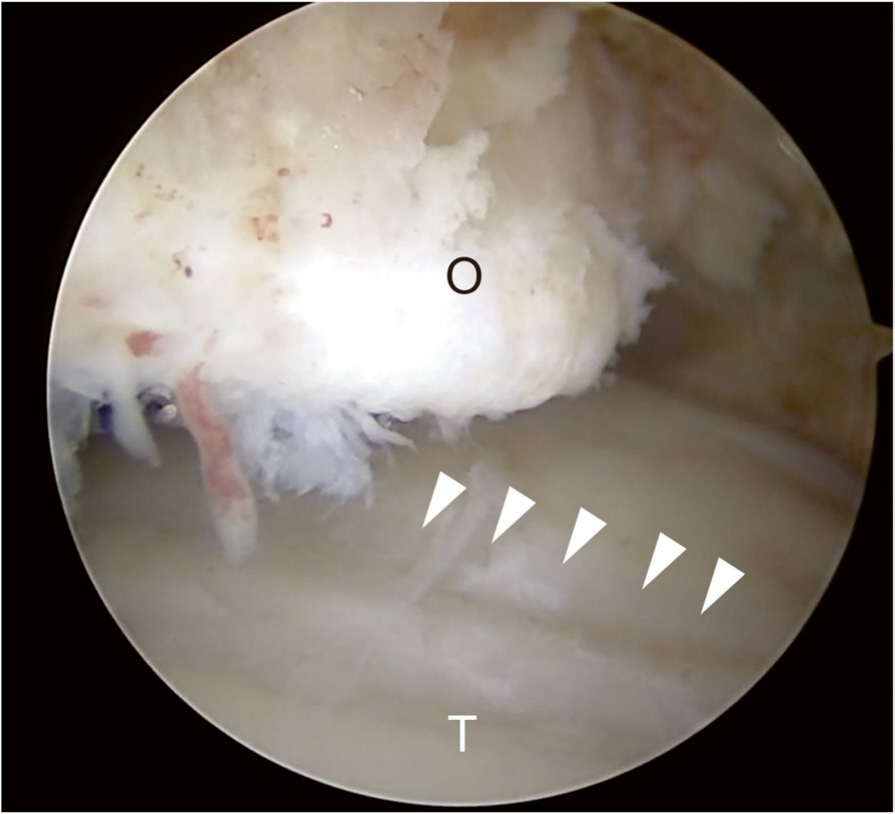
Arthroscopic findings of the ankle joint. Tram-track lesions (arrowheads) are longitudinal cartilage lesions on the talar dome (T), of which position corresponds to that of tibial osteophytes (O). T, talus; O, osteophytes

As a surgical intervention for anterior ankle impingement syndrome, arthroscopic resection of osteophytes and abnormal soft tissue showed good clinical outcomes [5, 6]. In addition, the arthroscopic treatment allows athletes to return to sports early postoperatively [7, 8]. However, it has been suggested that the severity of cartilage lesions affects the long-term clinical outcome of arthroscopic treatment for anterior ankle impingement syndrome [6, 9, 10]. Regarding cartilage lesions in anterior ankle impingement syndrome, Kim et al. described the longitudinal lesion with variable width located in the talar dome as tram-track lesions [11] (Fig. 1). Repetitive scratching by tibial osteophytes is believed to cause these distinctive cartilage lesions [12]. A previous study reported that the severity of tram-track lesions correlates with osteophyte severity evaluated using the McDermott classification [13, 14]. However, the relationship between cartilage lesion severity and the detailed osteophyte morphology, such as size and protrusion into joint space, has not been clarified. Therefore, the present study aimed to describe the frequency and severity of tram-track lesions in anterior ankle impingement syndrome in athletes and evaluate the association between osteophyte morphology and cartilage lesion severity.

## Methods

### Study design

This was a cross-sectional study at two institutions: Hanna Central Hospital, Nara, Japan and the Department of Orthopedic Surgery and Rehabilitation, Hachioji Sports Orthopedic Clinic, Tokyo, Japan.

### Participants

We retrospectively evaluated consecutive athletes who underwent arthroscopic osteophyte resection for anterior ankle impingement syndrome between January 2017 and March 2021. The indication for surgery was persistent symptoms of anterior ankle impingement syndrome, refractory to conservative treatment. Athletes with a history of ankle fracture or previous ankle surgery, those with incomplete study data, and those with findings of joint space narrowing on plain radiographs were excluded. This is the first study on this topic; therefore, we did not add other factors such as age, type of sports, and foot posture to our exclusion criteria and tended to be more inclusive. The following baseline characteristics data were collected from medical charts: sex, age, height, body weight, and affected side.

**Fig. 2:**
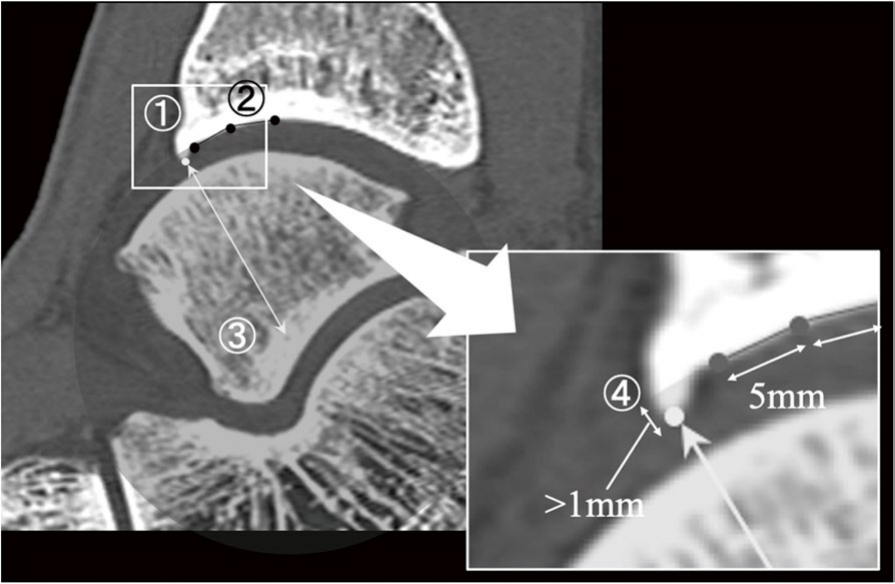
First, we marked a dot on the anterior tip of the distal tibia's articular surface on the sagittal CT view using freeware Image J. Second, we marked two more dots at 5 mm intervals from the first dot on the articular surface. Third, we calculated the coordinate of the center of a circle passing through these three dots, which correspond to the morphology of the anterior part of the tibial articular surface. Lastly, we marked a dot on the osteophyte tip and calculated the distance between the circle’s center and the dot on the osteophyte tips from their coordinates. If the distance is shorter than the circle’s radius by > 1 mm, osteophyte protrusion is confirm to be present

By reviewing the arthroscopic findings from a database, the presence of the tram-track lesions was assessed, and the cartilage lesion severity of tram-track lesions was graded using the International Cartilage Repair Society (ICRS) cartilage injury classification [15]. We measured the length from the anterior tip of the distal tibia’s articular surface to the osteophyte’s tip on each sagittal slice of computed tomography (CT). Their maximum value was defined as the osteophyte size. We also assessed the presence of spur protrusion into joint space using the following procedure. First, we marked a dot on the anterior tip of the distal tibia’s articular surface on the sagittal CT view using freeware Image J [16]. Second, we marked two more dots at 5 mm intervals from the first dot on the articular surface. Third, we calculated the coordinate of the center of a circle passing through these three dots, which correspond to the morphology of the anterior part of the tibial articular surface. Lastly, we marked a dot on the osteophyte tip and calculated the distance between the circle’s center and the dot on the osteophyte tips from their coordinates. If the distance is shorter than the circle’s radius by > 1 mm, osteophyte protrusion is confirmed to be present and defined as a protrusion type (Fig. 2). Considering this, we divided athletes into two groups by the presence or absence of osteophyte protrusion into joint space and compared their ICRS grade and osteophyte sizes. Post hoc power analysis was performed using G power [17].

### Statistical analysis

The correlation between the ICRS grade and osteophyte size was evaluated using Spearman’s rank correlation coefficient. The difference in ICRS grade concerning the presence or absence of osteophyte protrusion was evaluated using the Mann–Whitney U test. The Kruskal–Wallis test was used to examine the differences in ICRS grade and osteophyte size between rugby, soccer, and basketball, which have many participants. The baseline characteristics of the protrusion-type group and the non-protrusion-type group were compared using Fisher’s exact test, Mann–Whitney U test, unpaired t-test, and Pearson’s chi-square test. In addition, osteophyte size was compared between these two groups using the Mann–Whitney U test. Cohen’s Kappa coefficient was calculated to assess the inter- and intra-observer reliability of the cartilage injury grading. Intraclass correlation coefficients (ICC) were calculated to assess the inter- and intra-observer reliability of the measurement of osteophyte size. All analyses were performed using IBM SPSS Statistics version 29 (SPSS Inc., Chicago, IL, USA), referring to the results of the Shapiro–Wilk test. A *p*-value < 0.05 was considered statistically significant.

## Results

**Fig. 3:**
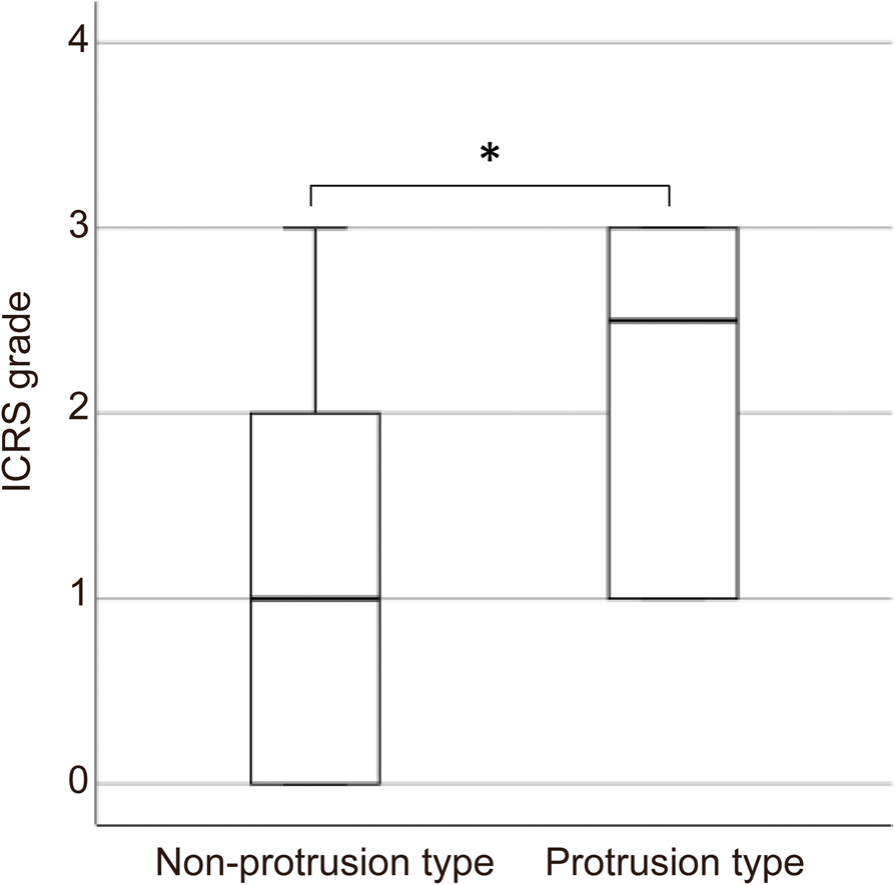
Boxplots showing the distribution of International Cartilage Repair Society (ICRS) grade according to the type of osteophytes. The boxes indicate the interquartile range. The lines within the boxes indicate the median (non-protrusion type: 1, protrusion type: 2.5). The whisker indicates the maximum value. The difference between the ICRS grade of the non-protrusion type group and that of the protrusion type group was significant (*p* = 0.008)

In the present study, 47 athletes consented to be enrolled initially. However, nine athletes with a history of ankle fracture or previous ankle surgery, three due to lack of arthroscopy data, and one with findings of joint space narrowing on plain radiograph were excluded. Eventually, the data of 34 athletes were included in the present study. All athletes had several months of conservative treatment with team trainers or at previous clinics, which failed to resolve symptoms before coming to our institutes. There were 27 male and 7 female athletes. The mean age was 24.6 years (range 17–39), the mean height was 174.8 cm (range 148–195), and the mean body weight was 74.9 kg (range 52.6–98). The affected side was the right in 19 athletes and the left in 15. Twenty-two athletes (64.7%) were professional, and the other 12 (35.3%) were student-athletes. These athletes were involved in sports, such as rugby (*n* = 10), soccer (*n* = 9), basketball (*n* = 6), volleyball (*n* = 3), baseball (*n* = 2), track and field (*n* = 2), hockey (*n* = 1), and badminton (*n* = 1). Participants’ clinical characteristics are shown in Table 1. Tram-track lesions were found in 26 (76.5%) of the 34 athletes. Arthroscopic findings of these athletes revealed that 8 athletes had grade 0, 7 had grade 1, 10 had grade 2, 9 had grade 3, and none had grade 4 cartilage lesions. Median osteophyte size was 3.95 mm (interquartile range, 2.8–7.2). Spearman’s rank correlation coefficient demonstrated a significant positive correlation between the ICRS grade and osteophyte size (r = 0.393, *p* = 0.021). There was no significant difference in osteophyte size and ICRS grade between the three sport types. Fourteen athletes (41.2%) had osteophyte protrusion into joint space. There were no significant differences between baseline characteristics of the non-protrusion type and protrusion type groups, except for sex (Table 2). All athletes in the protrusion-type group had tram-track lesions, and seven (50%) had ICRS grade 3. Seven of nine athletes with grade 3 cartilage lesions had osteophyte protrusion into the joint space (Table 3). The ICRS grade of the protrusion-type group was significantly higher than that of the non-protrusion-type group (*p* = 0.008) (Fig. 3). The effect size was 0.46. A post hoc power analysis demonstrated that the statistical power (two-tailed alpha, 0.05) to detect the observed difference in ICRS grade between the two groups using the Mann–Whitney U test was 81.3%. The osteophyte size in these two groups was not significantly different (*p* = 0.341). Cohen’s Kappa coefficient of the ICRS grade indicated almost perfect intra-observer reliability (κ = 0.925, *p* < 0.001) and substantial inter-observer reliability (κ = 0.667, *p* < 0.001) [18]. The intra- and inter-observer reliability of osteophyte size measurement were excellent, with an ICC of 0.99 (*p* = 0.00) and 0.98 (*p* < 0.01), respectively.

**Table 1:** Participant characteristics

Sex	
Male, n (%)	27 (79.4)
Female, n (%)	7 (20.6)
Age, mean (range)	24.6 (17–39)
Height (cm), mean (range)	174.8 (148.0–195.0)
Body weight (kg), mean (range)	74.9 (52.6–98.0)
Body mass index (kg/m^2^), mean (range)	24.4 (17.1–32.0)
Affected side	
Right, n (%)	19 (55.9)
Left, n (%)	15 (44.1)
Level of sports activity	
Student athlete, n (%)	12 (35.3)
Professional athlete, n (%)	22 (64.7)
Sport	
Rugby, n (%)	10 (29.4)
Soccer, n (%)	9 (26.5)
Basketball, n (%)	6 (17.6)
Volleyball, n (%)	3 (8.8)
Baseball, n (%)	2 (5.9)
Track and field, n (%)	2 (5.9)
Hockey, n (%)	1 (2.9)
Badminton, n (%)	1 (2.9)

*n* Number

**Table 2:** Baseline characteristics of the non-protrusion and protrusion type groups

	Non-protrusion (*n* = 20)	Protrusion (*n* = 14)	*P* value
Sex (male/female), n	13/7	14/0	0.014*
Age, median (interquartile range)	22.0 (20.0–27.6)	27.5 (22.0–30.3)	0.148
Height (cm), mean ± S.D.	173.7 ± 10.9	176.4 ± 8.1	0.432
Body weight (kg), mean ± S.D.	73.6 ± 14.7	76.6 ± 11.2	0.535
Body mass index (kg/m^2^), mean ± S.D.	24.3 ± 3.6	24.5 ± 2.5	0.829
Affected side (R/L), n	12/8	7/7	0.563

Fisher’s exact test was used for sex analysis. Mann-Whitney U test was used for age. Unpaired t-test was used for height, body weight, and body mass index. Pearson’s chi-square test was used for affected side. *n* Number, *S.D* Standard deviation

*Where at significant at *p* < .05 for Fisher's exact test

**Table 3:** Distribution of International Cartilage Repair Society (ICRS) grades according to the presence or absence of protrusion of osteophyte protrusion into the joint space in 34 patients

	ICRS grade					Total
	0	1	2	3	4	
Non-protrusion, n	8	3	7	2	0	20
Protrusion, n	0	4	3	7	0	14
Total, n	8	7	10	9	0	34

ICRS grade 0 indicates absence of tram track lesions. *n* Number

## Discussion

In the present study, athletes with anterior ankle impingement syndrome had frequent tram-track lesions, and more than half had ICRS grades 2 and 3 cartilage injuries. Furthermore, the most critical finding in the present study was that the osteophyte size and protrusion into the joint space were related to the severity of tram-track lesions.

The frequency of tram-track lesions in the present study (76.5%) was higher than that reported in previous studies (8.8% and 31.6%) [11, 13]. Regarding the severity of tram-track lesions, we compared the frequency of ICRS grade 3 in the present study to that of Outerbridge classification grade 4, almost equivalent to ICRS grade 3 in a previous study [13]. The result showed that the frequency of ICRS grade 3 (26.5%) in the present study was higher than that of Outerbridge grade 4 (17.5%) in a previous study [13]. A change in subject characteristics can cause these differences; however, the difference in the cartilage lesion evaluation can also affect the results. The subjects of the previous study consisted of four professional athletes (7.0%), 31 recreational athletes (41.3%), and 22 nonparticipants in sports (38.6%) [13]. However, the subjects of the present study consisted of 22 professional athletes (64.7%) and 12 student-athletes (35.3%); this shows that sports activity levels can affect the frequency and severity of cartilage lesions of tram-track lesions. In other words, athletes with anterior ankle impingement syndrome can have relatively severe tram-track lesions.

We could not find significant differences in osteophyte size and ICRS grade between the three types of sports in the present study; however, we believe that by conducting evaluations that include more sports and more participants, we will be able to clarify the differences in the impact of each sport on anterior ankle impingement syndrome. In addition, the present study’s main finding was that the ICRS grade in the protrusion-type group was higher than that in the non-protrusion-type group. The proportion of males was significantly higher in the protrusion-type group than in the non-protrusion-type group. In fact, all female athletes were in the non-protrusion-type group. As for differences between males and females in terms of anterior ankle impingement syndrome, it has been suggested that females have a higher percentage of combined anterior ankle impingement with chronic instability than males [19]. In addition, it has also been reported that females have higher rates of traumatic ankle sprains associated with anterior ankle impingement than males [20]. These findings indicate a possibility of sex-related differences in the mechanism of presenting symptoms of anterior ankle impingement. Considering the high incidence of anterior/anterolateral synovitis in patients with chronic lateral ankle instability [21], female athletes may present symptoms and undergo surgery in the earlier stage of anterior ankle impingement syndrome without developing larger or protruding osteophyte than male athletes.

Contrarily, male athletes may present symptoms in the relatively late stage of anterior ankle impingement syndrome with large or protruding osteophyte formation. Information regarding ankle instability was unavailable in the present study; however, the age of female participants was significantly low, and the osteophyte size of female participants was significantly small. These differences may be caused by sex-related differences such as ankle stability, dynamic postural control [22], and coordination and variability among foot joints [23]. Furthermore, a previous study suggested that female patients have higher rates of chondral injury in anterior ankle impingement syndrome [10]. No other studies have reported the relationship between sex and cartilage injury in anterior ankle impingement syndrome. Therefore, we considered that the difference in sex ratio between the two groups is unlikely to make us overestimate the effect of protrusion on cartilage lesion severity. There were no significant differences in the osteophyte size of these two groups. These findings showed that osteophyte morphology influences the severity of tram-track lesions independent of osteophyte size. In the present study, 53.8% of athletes in the protrusion-type group had ICRS grade 3 tram-track lesions. In addition, seven of nine athletes with grade 3 cartilage lesions had osteophyte protrusion into joint space. It has been suggested that the severity of cartilage lesions affects the long-term clinical outcome of arthroscopic treatment for anterior ankle impingement syndrome [6, 9]. Based on these findings, osteophyte protrusion should be assessed when considering an indication of arthroscopic treatment for anterior ankle impingement syndrome in athletes. The tram-track lesions can be detected by 3.0-tesla magnetic resonance imaging [24]; however, the information demonstrated in the present study will be useful for decision-making. We believe that if athletes experiencing anterior ankle impingement syndrome have protrusion-type osteophytes, arthroscopic resection can help them continue their careers with good performance for an extended time.

This study had some limitations. First, the number of subjects was small. Second, we did not assess ankle joint instability, clinical score, foot posture, and years of experience in the athletic field, which could have affected the degree of cartilage lesions. Therefore, considering these factors, a large cohort study is required to evaluate the relationship between osteophyte morphology and the severity of tram-track lesions in athletes.

## Conclusions

This is the first study to describe the frequency and severity of tram-track lesions in anterior ankle impingement syndrome in athletes and evaluate the association between osteophyte morphology and tram-track lesion severity. In the present study, athletes with anterior ankle impingement syndrome had more frequent and severe tram-track lesions than those previously reported. The severity of cartilage lesions was associated with the size and protrusion of osteophytes. Protrusion-type osteophyte was more common in male athletes. Further studies on many athletes across multiple sports are necessary, with additional information such as ankle instability, foot posture, and athletic history to elucidate the detailed impact of osteophyte morphology and sports on cartilage lesions.

## Data Availability

All data generated or analyzed during this study are included in this published article and its supplementary data.

## Abbreviations

ICRS: International cartilage repair society
CT: Computed tomography
ICC: Intraclass correlation coefficients
